# New characterization of dihydroergotamine receptor pharmacology in the context of migraine: utilization of a β-arrestin recruitment assay

**DOI:** 10.3389/fneur.2023.1282846

**Published:** 2023-11-21

**Authors:** Lisa McConnachie, Peter J. Goadsby, Robert E. Vann, Sutapa Ray, Stephen B. Shrewsbury, Sheena K. Aurora

**Affiliations:** ^1^Priovant Therapeutics, New York, NY, United States; ^2^Impel Pharmaceuticals, Seattle, WA, United States; ^3^NIHR King’s Clinical Research Facility, King’s College London, London, United Kingdom; ^4^Department of Neurology, David Geffen School of Medicine at University of California-Los Angeles, Los Angeles, CA, United States

**Keywords:** migraine, dihydroergotamine, receptor, pharmacology, binding

## Abstract

**Introduction:**

Dihydroergotamine mesylate (DHE) is an established effective acute therapy for migraine and is often characterized by its broad receptor pharmacology. Knowledge of DHE pharmacology largely comes from studies employing older methodologies.

**Objective:**

To assess DHE receptor activity using high-throughput methods to screen for functional ß-arrestin activity at G protein–coupled receptors (GPCRs).

**Methods:**

Functional receptor activities of DHE and sumatriptan succinate (both 10 μM) were screened against 168 GPCRs using the gpcrMAX assay. Agonist and antagonist effects were considered significant if receptor activity was >30% or inhibited by >50%, respectively. Radiolabeled ligand binding assays were performed for DHE (0.01–300 nM for 5-HT_3_ and _4E_; 0.3–10,000 nM for 5-HT_1B_, α-adrenergic_2B_ [i.e., α_2B_-adrenoceptor], D_2_, and D_5_) to assess specific binding to select receptors.

**Results:**

DHE (10 μM) exhibited agonist activity at α-adrenergic_2B_, CXC chemokine receptor 7 (CXCR7), dopamine (D)_2/5_, and 5-hydroxytryptamine (5-HT)_1A/1B/2A/2C/5A_ receptors and antagonist activity at α-adrenergic_1B/2A/2C_ (i.e., α_1B/2A/2C_-adrenoceptors), calcitonin receptor–receptor activity modifying protein 2 (CTR-RAMP2) or amylin 2 (AMY_2_), D_1/3/4/5_, and 5-HT_1F_ receptors. Sumatriptan succinate (10 μM) exhibited agonist activity at the 5-HT_1B/1E/1F/5A_ receptors. DHE demonstrated a half-maximal inhibitory concentration (IC_50_) of 149 nM at the 5-HT_1F_ receptor and a half-maximal effective concentration (EC_50_) of 6 μM at the CXCR7 receptor. DHE did not bind to the 5-HT_3_ receptor at concentrations up to 300 nM and bound poorly to 5-HT_4E_ and D_5_ receptors (IC_50_ of 230 and 370 nM, respectively). DHE bound strongly to the D_2_, 5-HT_1B_, and α-adrenergic_2B_ receptors (IC_50_ of 0.47, 0.58, and 2.8 nM, respectively).

**Conclusion:**

By using a high-throughput β-arrestin recruitment assay, this study confirmed the broad receptor profile of DHE and provided an update on DHE receptor pharmacology as it relates to migraine.

## Introduction

Migraine pathophysiology is complex, involving multiple regions of the brain, neurotransmitters, neuropeptides, ion channels, and numerous receptor pathways (1–3). An increased understanding of this pathophysiology has led to the development of novel therapeutic targets for the treatment of migraine. The development of narrowly targeted therapies for the acute treatment of migraine began in the 1980s with the advent of triptans, which are 5-hydroxytryptamine-_1B/1D_ (5-HT_1B/1D_) receptor agonists, and continued with the recent approval of gepants, which are calcitonin gene-related peptide (CGRP) receptor antagonists, and lasmiditan, which is a 5-HT_1F_ receptor agonist (4–6). By selectively targeting mediators and mechanisms shown to be involved in migraine pathophysiology, pharmacologic agents can potentially alleviate migraine symptoms, including pain, while minimizing unwanted tolerability concerns in patients (7). However, the potential benefit in the interplay between different pathways may then be left unaddressed. A comprehensive description of receptor binding, specifically which key receptors in migraine pathophysiology are activated and how various pathways may influence each other, is critical in understanding the presence or absence of clinical efficacy of migraine therapies.

Dihydroergotamine mesylate (DHE) is a familiar molecule among headache specialists and has been a mainstay for difficult-to-treat migraine, offering patients single-dose efficacy in a rapid and consistent manner (8–10). Over the course of many decades, several review articles on DHE pharmacology have been published, each suggesting that the efficacy of DHE may be attributed to its broad receptor coverage, which includes serotonergic, adrenergic, and dopaminergic receptor activity (11, 12); however, much of our understanding of DHE receptor pharmacology from these review articles are results from studies using older methodologies, performed decades ago. The most recent study was performed by Cook and colleagues, who sought to determine whether differences in the binding and functional activity of intravenous (IV) DHE and an orally inhaled DHE product, MAP0004, could explain the improved adverse event profile observed with MAP0004 (9, 12). A high DHE concentration (5 μM) was used to screen against 65 receptors, ion channels, and enzymes. Using DHE concentrations corresponding to the maximum plasma concentration (C_max_) of 1 mg of IV DHE (53 ng/mL [~0.091 μM]), 4 inhalations of MAP0004 (systemic equivalent to 0.88 mg; 4.3 ng/mL [~0.007 μM]), and 2 inhalations of MAP0004 (systemic equivalent to 0.44 mg; 1.3 ng/mL [~0.002 μM]), a customized radioligand receptor binding screening profile was then performed to determine binding activity, and functional receptor activity was determined with *in vitro* techniques using several signaling pathways. The receptor binding profile for IV DHE was more extensive compared to both MAP0004 doses (Table 1). With regard to functional receptor binding for IV DHE and MAP0004, functional agonist activity of DHE was demonstrated at the 5-HT_1A/1B/1D_ receptors. Functional antagonist activity at the dopamine (D)_2_ receptor was reported for IV DHE and 4 MAP0004 inhalations and at the 5-HT_2A_ receptor for IV DHE. Functional antagonist activity was also determined at the α-adrenergic_1A/2A/2B_ receptors (i.e., α_1A/2A/2B_-adrenoceptors) for IV DHE, with reduced or absent antagonism for both MAP0004 doses. These results demonstrated that the interaction profile with regard to specific receptors was concentration dependent (12).

**Table 1:** Previously published receptor radioligand binding of IV DHE compared with MAP0004 (12).

Receptor	IV DHE (53 ng/mL)	MAP0004 (4.3 ng/mL)	MAP0004 (1.3 ng/mL)
5-HT_1A_	X	X	X
5-HT_1B_	X	X	X
5-HT_1D_	X		
5-HT_2A_	X		
5-HT_2C_	X		
5-HT_3_			
5-HT_4_			
5-HT_5A_	X		
5-HT_6_	X	X	
5-HT_7_	X	X	
α-adrenergic_1_	X		
α-adrenergic_2A_	X	X	
α-adrenergic_2B_	X	X	
α-adrenergic_2C_	X	X	
β-adrenergic			
D_1_			
D_2S_	X		
D_3_	X	X	X

Table adapted from Cook et al 2009. Receptor binding was measured as percent receptor binding, where > 50% was considered to be an active response and < 20% was considered to be an inactive response. An X denotes active binding.

5-HT, 5-hydroxytryptamine; D, dopamine; DHE, dihydroergotamine mesylate; IV, intravenous.

There have been advances in receptor assay methodology since the study by Cook and colleagues (12). Traditional receptor binding studies, which often require secondary functional activity assays to establish agonism or antagonism, are useful tools to determine the activity of a drug at a specific receptor (12, 13). A more updated approach for assessing receptor activity includes high-throughput methods assessing reporter protein activity, such as ß-arrestin, to screen rapidly for ligand activity, as opposed to binding, at various G protein–coupled receptors (GPCRs) (14). ß-arrestin is a ubiquitously expressed protein that plays an important role in cell signaling, and recruitment of ß-arrestin following ligand binding to GPCRs is well characterized (14–18). Several high-throughput screening approaches that rely on the detection of ß-arrestin recruitment (14, 17) to evaluate unknown ligand binding to GPCRs have been developed (eg, the PRESTO-Tango platform (19) or the PathHunter® Assay (20)) and can be performed relatively rapidly and efficiently. One advantage of these assays is that the activity proximal to, rather than downstream of, specific G protein activation is measured, which may minimize false positives resulting from off-target effects due to downstream signaling cascades (21). Results from these assays provide a comprehensive assessment of binding and activity and are useful for understanding the potential of both on- and off-target effects (14). A potential disadvantage of these approaches is that they are typically performed using one concentration of the test ligand, and follow-up evaluations are required should positive results be obtained in the screening assay. The objective of the present study was to build on previous work to further update our understanding of DHE receptor activity and provide a relevant clinical context for the mechanism of action of DHE as an acute therapy for migraine.

## Methods

### *In vitro* screening for functional receptor activity of DHE and sumatriptan succinate

Functional receptor activity of DHE and sumatriptan succinate was screened against 168 GPCRs with the gpcrMAX assay panel, which utilizes the PathHunter β-arrestin enzyme fragment complementation technology (Eurofins DiscoverX; Fremont, CA; Table 2). The gpcrMAX panel evaluates ß-arrestin recruitment and was carried out in agonist and antagonist modes using 10 μM each of DHE and sumatriptan succinate. The human plasma C_max_ of DHE depends on the dose, formulation, and route of administration. A phase 1 study assessed the pharmacokinetics of 1.45 mg of INP104 (DHE delivered by Precision Olfactory Delivery; Impel Pharmaceuticals, Seattle, WA), 1.0 mg of IV DHE, and 2.0 mg of DHE nasal spray (Migranal®, Bausch Health Companies, Inc. or its affiliates, Bridgewater, NJ). Human plasma C_max_ was ~2 nM (1.3 ng/mL), ~24 nM (14.2 ng/mL), and ~ 0.5 nM (0.3 ng/mL) for INP104, IV DHE, and DHE nasal spray, respectively (24, 25). Similarly, the C_max_ was ~173–182 nM (51–53.8 ng/mL), ~54 nM (16 ng/mL), and ~ 240–245 nM (71–72.4 ng/mL) for 100 mg of oral sumatriptan, 20 mg of sumatriptan nasal spray, and 6 mg of subcutaneous sumatriptan, respectively (26–28).

**Table 2:** Receptors included in the screening for functional receptor activity of DHE (10 μM) (22).

Family name	Human gene	Common name
5-Hydroxytryptamine receptors	*HTR1A*	5-HT_1A_ receptor
	*HTR1B*	5-HT_1B_ receptor
	*HTR1F*	5-HT_1F_ receptor
	*HTR2A*	5-HT_2A_ receptor
	*HTR2C*	5-HT_2C_ receptor
	*HTR5A*	5-HT_5A_ receptor
	*HTR1E*	5-HT_1E_ receptor
Acetylcholine receptors	*CHRM1*	M_1_ receptor
	*CHRM2*	M_2_ receptor
	*CHRM3*	M_3_ receptor
	*CHRM4*	M_4_ receptor
	*CHRM5*	M_5_ receptor
Adenosine receptors	*ADORA3*	A_3_ receptor
Adrenoceptors	*ADRA1B*	α-adrenergic_1B_ (α_1B_-adrenoceptor)
	*ADRA2A*	α-adrenergic_2A_ (α_2A_-adrenoceptor)
	*ADRA2B*	α-adrenergic_2B_ (α_2B_-adrenoceptor)
	*ADRA2C*	α-adrenergic_2C_ (α_2C_-adrenoceptor)
	*ADRB1*	β-adrenergic_1_ (β_1_-adrenoceptor)
	*ADRB2*	β-adrenergic_2_ (β_2_-adrenoceptor)
Angiotensin receptor	*AGTR1*	AT_1_ receptor
Apelin receptor	*AGTRL1 (APLNR)*	APJ (Apelin receptor)
Bombesin receptors	*BRS3*	BB_3_ receptor
	*GRPR*	BB_2_ receptor
	*NMBR*	BB_1_ receptor
Bradykinin receptors	*BDKRB1*	B_1_ receptor
	*BDKRB2*	B_2_ receptor
Calcitonin receptors	*CALCR*	CT receptor
	*CALCRL-RAMP1 (NA)*	CGRP receptor
	*CALCRL-RAMP2 (NA)*	AM_1_ receptor
	*CALCRL-RAMP3 (NA)*	AM_2_ receptor
	*CALCR-RAMP2 (NA)*	AMY_2_ receptor
	*CALCR-RAMP3 (NA)*	AMY_3_ receptor
Cannabinoid receptors	*CNR1*	CB_1_ receptor
	*CNR2*	CB_2_ receptor
Chemerin receptor	*CMKLR1*	CMKLR1 (Chemerin receptor 1)
Chemokine receptors	*CCR1*	CCR1
	*CCR10*	CCR10
	*CCR2*	CCR2
	*CCR3*	CCR3
	*CCR4*	CCR4
	*CCR5*	CCR5
	*CCR6*	CCR6
	*CCR7*	CCR7
	*CCR8*	CCR8
	*CCR9*	CCR9
	*CX_3_CR1*	CX_3_CR1
	*CXCR1*	CXCR1
	*CXCR2*	CXCR2
	*CXCR3*	CXCR3
	*CXCR4*	CXCR4
	*CXCR5*	CXCR5
	*CXCR7*	CXCR7
Cholecystokinin receptors	*CCKAR*	CCK_1_ receptor
	*CCKBR*	CCK_2_ receptor
Class A orphans	*EBI2 (GPR183)*	GPR183
	*GPR1 (CMKLR2)*	GPR1 (Chemerin receptor 2)
	*GPR119*	GPR119
	*GPR35*	GPR35
	*MRGPRX1*	MRGPRX1
	*MRGPRX2*	MRGX2
Complement peptide receptors	*C5AR1*	C5A receptor (C5_a1_ receptor)
	*C5L2 (C5AR2)*	C5L2 receptor (C5_a2_ receptor)
Corticotropin-releasing factor receptors	*CRHR1*	CRF1 receptor
	*CRHR2*	CRF2 receptor
Dopamine receptors	*DRD1*	D_1_ receptor
	*DRD2L*	D_2L_ receptor
	*DRD2S*	D_2S_ receptor
	*DRD3*	D_3_ receptor
	*DRD4*	D_4_ receptor
	*DRD5*	D_5_ receptor
Endothelin receptors	*EDNRA*	ET_A_ receptor
	*EDNRB*	ET_B_ receptor
Formylpeptide receptors	*FPR1*	FPR1
	*FPRL1 (FPR2)*	FPR2/ALX
Free fatty acid receptors	*FFAR1*	FFA1 receptor
	*GPR120 (FFAR4)*	FFA4 receptor
Galanin receptors	*GALR1*	GALR_1_ receptor (GAL_1_ receptor)
	*GALR2*	GALR_2_ receptor (GAL_2_ receptor)
Ghrelin receptor	*GHSR*	Ghrelin receptor
Glucagon receptors	*GCGR*	Glucagon receptor
	*GIPR*	GIP receptor
	*GLP1R*	GLP-1 receptor
	*GLP2R*	GLP-2 receptor
	*SCTR*	Secretin receptor
Glycoprotein hormone receptors	*FSHR*	FSHR receptor (FSH receptor)
	*LHCGR*	LH receptor
	*TSHR(L) (TSHR)*	TSH receptor
Histamine receptors	*HRH1*	H_1_ receptor
	*HRH2*	H_2_ receptor
	*HRH3*	H_3_ receptor
	*HRH4*	H_4_ receptor
Hydroxycarboxylic acid receptors	*GPR109A (HCAR2)*	HCA_2_ receptor
	*GPR109B (HCAR3)*	HCA_3_ receptor
Kisspeptin receptor	*KISS1R*	Kisspeptin receptor
Leukotriene receptors	*LTB4R*	BLT_1_ receptor
	*OXER1*	OXE receptor
Lysophospholipid (LPA) receptors	*EDG4 (LPAR2)*	LPA_2_ receptor
	*EDG7 (LPAR3)*	LPA_3_ receptor
	*GPR92 (LPAR5)*	GPR92 receptor (LPA_5_ receptor)
Lysophospholipid (S1P) receptors	*EDG1 (S1PR1)*	S1P_1_ receptor
	*EDG3 (S1PR3)*	S1P_3_ receptor
	*EDG5 (S1PR2)*	S1P_2_ receptor
	*EDG6 (S1PR4)*	S1P_4_ receptor
Melanin-concentrating hormone receptors	*MCHR1*	MCH_1_ receptor
	*MCHR2*	MCH_2_ receptor
Melanocortin receptors	*MC1R*	MC_1_ receptor
	*MC3R*	MC_3_ receptor
	*MC4R*	MC_4_ receptor
	*MC5R*	MC_5_ receptor
Melatonin receptor	*MTNR1A*	MT_1_ receptor
Motilin receptor	*MLNR*	Motilin receptor
Neuromedin U receptor	*NMU1R*	NMU1 receptor
Neuropeptide B and W receptors	*NPBWR1*	NPBW1 receptor
	*NPBWR2*	NPBW2 receptor
Neuropeptide FF and AF receptor	*NPFFR1*	NPFF1 receptor
Neuropeptide S receptor	*NPSR1b (NPSR1)*	NPS receptor
Neuropeptide Y receptors	*NPY1R*	Y_1_ receptor
	*NPY2R*	Y_2_ receptor
	*PPYR1 (NPY4R)*	Y_4_ receptor
Neurotensin receptor	*NTSR1*	NTS_1_ receptor
Opioid receptors	*OPRD1*	δ receptor
	*OPRK1*	κ receptor
	*OPRL1*	NOP receptor
	*OPRM1*	μ receptor
Orexin receptors	*HCRTR1*	OX_1_ receptor
	*HCRTR2*	OX_2_ receptor
P2Y receptors	*P2RY1*	P2Y_1_ receptor
	*P2RY11*	P2Y_11_ receptor
	*P2RY12*	P2Y_12_ receptor
	*P2RY2*	P2Y_2_ receptor
	*P2RY4*	P2Y_4_ receptor
	*P2RY6*	P2Y_6_ receptor
Parathyroid hormone receptors	*PTHR1 (PTH1R)*	PTH1 receptor
	*PTHR2 (PTH2R)*	PTH2 receptor
Peptide P518 receptor	*GPR103 (QRFPR)*	QRFPR receptor
Platelet-activating factor receptor	*PTAFR*	PAF receptor
Prokineticin receptors	*PROKR1*	PKR_1_ receptor
	*PROKR2*	PKR_2_ receptor
Prolactin-releasing peptide receptor	*PRLHR*	PRRP receptor (PrRP receptor)
Prostanoid receptors	*CRTH2 (PTGDR2)*	PTGDR2 receptor (DP_2_ receptor)
	*PTGER2*	EP_2_ receptor
	*PTGER3*	EP_3_ receptor
	*PTGER4*	EP_4_ receptor
	*PTGFR*	FP receptor
	*PTGIR*	IP1 receptor (IP receptor)
	*TBXA2R*	TP receptor
Protease activated receptors	*F2R*	PAR1
	*F2RL1*	PAR2
	*F2RL3*	PAR4
Relaxin family peptide receptor	*RXFP3*	RXFP3
Somatostatin receptors	*SSTR1*	SST_1_ receptor
	*SSTR2*	SST_2_ receptor
	*SSTR3*	SST_3_ receptor
	*SSTR5*	SST_5_ receptor
Tachykinin receptors	*TACR1*	NK_1_ receptor
	*TACR2*	NK_2_ receptor
	*TACR3*	NK_3_ receptor
Thyrotropin-releasing hormone receptor	*TRHR*	TRH_1_ receptor
Urotensin receptor	*UTR2 (UTS2R)*	UT receptor
Vasopressin and oxytocin receptors	*AVPR1A*	V_1A_ receptor
	*AVPR1B*	V_1B_ receptor
	*AVPR2*	V_2_ receptor
	*OXTR*	OT receptor
VIP and PACAP receptors	*ADCYAP1R1*	PAC_1_ receptor
	*VIPR1*	VPAC_1_ receptor
	*VIPR2*	VPAC_2_ receptor

This table refers to receptor nomenclature at the time of assay performance. Information in parentheses refers to any updates in nomenclatures per IUPHAR guidelines (23).

DHE, dihydroergotamine mesylate; IUPHAR, International Union of Basic and Clinical Pharmacology; LPA, lysophosphatidic acid; NA, not applicable; S1P, sphingosine-1 phosphate; PACAP, pituitary adenylate cyclase-activating peptide; VIP, vasoactive intestinal peptide.

PathHunter cell lines were expanded from freezer stocks according to standard procedures and cells were seeded in a total volume of 20 μL into white-walled, 384-well microplates in duplicate and incubated at 37°C prior to testing. For agonist activity, cells expressing the various receptors were incubated with 10 μM DHE mesylate or 10 μM sumatriptan succinate. Intermediate dilution (1% vehicle concentration) of sample stocks was performed to generate a 5× sample in assay buffer, of which 5 μL was added to the cells and incubated for 90 or 180 min at 37°C or room temperature, depending on the specific receptor, as established by manufacturer optimization protocols (see Supplementary Table 1). For antagonist activity, cells were preincubated with an antagonist for 30 min, followed by an agonist challenge at 80% of the maximal effective concentration (EC_80_). Intermediate dilution of sample stocks was performed to generate 5× sample in assay buffer, of which 5 μL was added to cells and incubated at 37°C or room temperature for 30 min. This was followed by an addition of 5 μL of 6× EC_80_ agonist in assay buffer to the cells, which were incubated at 37°C or room temperature for 90 or 180 min. Assay signal for agonist and antagonist modes was generated through a single addition of 12.5 or 15 μL (50% v/v) of PathHunter Detection reagent cocktail, followed by a 1-h incubation at room temperature. Further experimental details can be found in the Supplementary File. Microplates were read with a PerkinElmer EnVision (Shelton, CT) instrument for chemiluminescent signal detection. The gpcrMAX panel does not include a cell line expressing human 5-HT_1D._

### Radioligand competition binding assays

Because the gpcrMAX panel assessed ß-arrestin recruitment with a single concentration of DHE, a range of concentrations was used to determine DHE binding to 4 select GPCRs (5-HT_1B,_ α-adrenergic_2B_, D_2_, and D_5_). The 5-HT_3_ and 5-HT_4E_ receptors were also evaluated because they had not been evaluated in the gpcrMAX assay. All assays were performed in duplicate (ie, 2 replicates per assay, per standard manufacturer protocol).

#### 5-HT_3_

Binding of DHE to the human 5-HT_3_ receptor was evaluated via a radioligand binding assay in transfected human recombinant HEK-293 cells and performed by Eurofins Panlabs Discovery Services (New Taipei City, Taiwan). Cell membrane homogenates (30 μg protein) were incubated for 60 min at 25°C with 0.69 nM [^3^H]GR-65630 in the absence or presence of DHE in a buffer containing 50 mM Tris–HCl (pH 7.4), 5 mM MgCl_2_, and 1 mM ethylenediaminetetraacetic acid (29). The experiment was conducted in a 96-well plate format with 200 μL total volume and 8 concentrations of DHE, ranging from 0.01 to 300 nM. This concentration range was selected to cover the human plasma C_max_ of DHE administered by multiple routes. Nonspecific binding was determined in the presence of 10 μM MDL 72222. Following incubation, the samples were filtered rapidly under vacuum through glass fiber filters (GF/B, Packard; Kennesaw, GA) presoaked with 0.3% polyethyleneimine and rinsed several times with ice-cold 50 mM Tris–HCl using a 96-sample cell harvester (UniFilter, Packard). The filters were dried and then counted for radioactivity in a scintillation counter (TopCount, Packard) using a scintillation cocktail (MicroScint 0, Packard). Results are expressed as percent inhibition of the control radioligand–specific binding. The standard reference compound was MDL 72222, which was tested in each experiment at several concentrations to obtain a competition curve from which its half-maximal inhibitory concentration (IC_50_) was calculated.

#### 5-HT_4E_

Binding of DHE to the human 5-HT_4E_ receptor was similarly evaluated via a radioligand binding assay in transfected human recombinant Chinese hamster ovary (CHO) cells and performed by Eurofins Cerep SA (Le Bois L’Evêque, France). Cell membrane homogenates (140 μg protein) were incubated for 60 min at 37°C with 0.3 nM [^3^H]GR 113808 in the absence or presence of DHE in a buffer containing 50 mM HEPES/Tris (pH 7.4) and 1 μM pargyline (30). The experiment was conducted in a 96-well plate format with 200 μL total volume, and 8 concentrations of DHE (0.01–300 nM) were evaluated. Nonspecific binding was determined in the presence of 100 μM serotonin. Following incubation, the same protocol as described above for 5-HT_3_ binding was employed. The standard reference compound for 5-HT_4E_ binding is serotonin.

#### 5-HT_1B_, α-adrenergic_2B_, dopamine (D)_2_, and D_5_

Binding of DHE to 5-HT_1B,_ α-adrenergic_2B_, D_2_, and D_5_ receptors was evaluated via a radioligand binding assay in human recombinant Chem-1 cells, CHO cells, HEK-293 cells, and GH4 cells, respectively, and performed by Eurofins Cerep SA. The incubation time was 60 min at room temperature (or 37°C for 5-HT_1B_) with [^3^H]RX 821002, [^3^H]7-OH-DPAT, [^3^H]SCH 23390, [^3^H]GR125743 for α-adrenergic_2B_, D_2_, D_5_, and 5-HT_1B_, respectively (31–34). Concentrations of DHE ranged from 0.3 to 10,000 nM. Nonspecific binding was determined in the presence of (−)epinephrine (100 μM), butaclamol (10 μM), SCH 23390 (10 μM), and serotonin (10 μM) for α-adrenergic_2B_, D_2_, D_5_, and 5-HT_1B_, respectively.

### Data analysis of functional receptor activity

DHE and sumatriptan succinate activity were analyzed using CBIS data analysis suite (ChemInnovation; San Diego, CA). Measurement of agonist and antagonist activity in the assay was calculated as percent activity of relative luminescence units (from positive control). Significance of agonist/antagonist activity was determined based upon prespecified criteria provided by Eurofins DiscoverX. In brief, receptor activity >30% was considered a significant agonist effect. Receptor inhibition >50% was considered a significant inhibitory effect. Please refer to the Supplementary File for more detail.

## Results

### *In vitro* screening for functional receptor activity of DHE and sumatriptan succinate

DHE (10 μM) exhibited agonist activity at α-adrenergic_2B_, CXC chemokine receptor 7 (CXCR7), D_2/5_, and 5-HT_1A/1B/2A/2C/5A_ receptors (Table 3). DHE (10 μM) exhibited antagonist activity at α-adrenergic_1B/2A/2C_ (i.e., α_1B/2A/2C_-adrenoceptors), calcitonin receptor–receptor activity modifying protein 2 (CTR-RAMP2) or amylin 2 (AMY_2_), D_1/3/4/5_, and 5-HT_1F_ receptors (Table 4). Sumatriptan succinate (10 μM) exhibited agonist activity at 5-HT_1B/1E/1F/5A_ receptors and no antagonist activity at any receptor tested (Table 5). In the initial screening, DHE exhibited fairly strong antagonist activity at the 5-HT_1F_ receptor and agonist activity at the CXCR7 receptor. Because of this, a more-thorough evaluation of ß-arrestin recruitment was performed to determine the activity of DHE at these receptors. The IC_50_ for DHE was 149 nM at the 5-HT_1F_ receptor, and the EC_50_ was 6 μM at the CXCR7 receptor.

**Table 3:** gpcrMAX agonist mode results for DHE.

Receptor	% Activity	Agonist control
α-adrenergic_2B_	88	UK 14,304
CXCR7	83	CXCL12
D_2L_	70	Dopamine
D_2S_	60	Dopamine
D_5_	57	Dopamine
5-HT_1A_	100	Serotonin
5-HT_1B_	52	Serotonin
5-HT_2A_	56	Serotonin
5-HT_2C_	76	Serotonin
5-HT_5A_	66	Serotonin

DHE (10 μM) was screened against 168 GPCRs. Receptor activity, as measured relative to a known receptor agonist, greater than 30% was considered a significant agonist effect. Receptors meeting this criterion are presented here.

5-HT, 5-hydroxytryptamine; CXCL12, chemokine (C-X-C motif) ligand 12; CXCR7, CXC chemokine receptor 7; D, dopamine; DHE, dihydroergotamine mesylate; GPCR, G protein–coupled receptor.

**Table 4:** gpcrMAX antagonist mode results for DHE.

Receptor	% Inhibition	Agonist control
α-adrenergic_1B_	95	Phenylephrine
α-adrenergic_2A_	115	UK 14,304
α-adrenergic_2C_	124	UK 14,304
AMY_2_	57	Calcitonin
D_1_	71	Dopamine
D_3_	91	Dopamine
D_4_	83	Dopamine
D_5_	54	Dopamine
5-HT_1F_	92	Serotonin

DHE (10 μM) was screened against 168 GPCRs in antagonist mode. Receptor inhibition, as measured by inhibition by a known receptor agonist, greater than 50% was considered a significant inhibitory effect. Receptors meeting this criterion are presented here.

5-HT, 5-hydroxytryptamine; AMY_2_, amylin 2; D, dopamine; DHE, dihydroergotamine mesylate; GPCR, G protein–coupled receptor.

**Table 5:** gpcrMAX agonist mode results for sumatriptan succinate.

Receptor	% Activity	Agonist control
5-HT_1B_	115	Serotonin
5-HT_1E_	51	Serotonin
5-HT_1F_	83	Serotonin
5-HT_5A_	48	Serotonin

Sumatriptan succinate (10 μM) was screened against 168 GPCRs. Receptor activity, as measured relative to a known receptor agonist, greater than 30% was considered a significant agonist effect. Receptors meeting this criterion are presented here.

5-HT, 5-hydroxytryptamine; GPCR, G protein–coupled receptor.

### Radioligand competition binding assays

**Figure 1:**
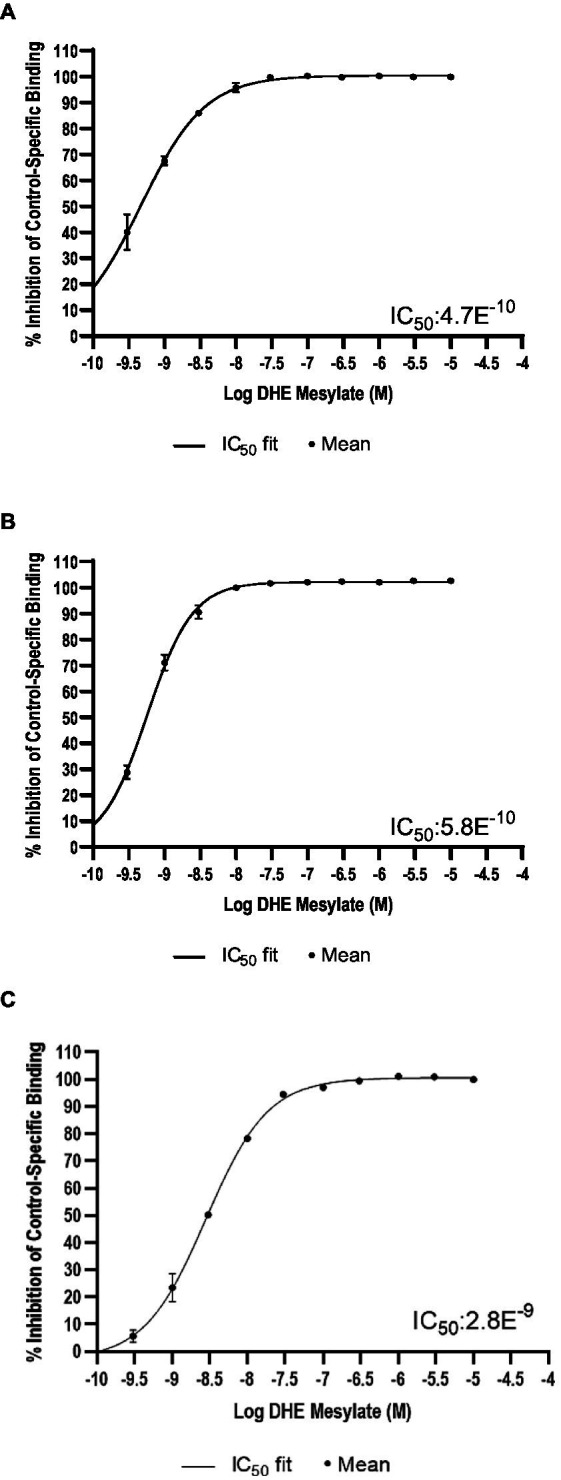
Radioligand binding results. Percent inhibition of radioligand binding to **(A)** the D_2_* receptor, **(B)** the 5-HT_1B_ receptor, and **(C)** the α-adrenergic_2B_ receptor in the presence of DHE. *Analysis was for D_2S_. 5-HT, 5-hydroxytryptamine; D, dopamine; DHE, dihydroergotamine mesylate; IC_50_, half-maximal inhibitory concentration.

DHE did not exhibit binding to the 5-HT_3_ receptor at concentrations up to 300 nM and bound poorly to 5-HT_4E_ and D_5_ receptors, with IC_50_ values of 230 and 370 nM, respectively (Table 6). DHE exhibited stronger binding to the D_2_, 5-HT_1B_, and α-adrenergic_2B_ receptors, with IC_50_ values of 0.47, 0.58, and 2.8 nM, respectively (Figure 1; Table 6).

**Table 6:** Radiolabeled ligand binding assay results for DHE.

Receptor	IC_50_ (nM)
5-HT_1B_	0.58
5-HT_3_	>300
5-HT_4E_	230
α-adrenergic_2B_	2.8
D_2_	0.47
D_5_	370

Membrane fractions of human recombinant cell lines each expressing the specific receptor were incubated in the presence of DHE (0.01–300 nM for 5-HT_4E_, 0.3–10,000 nM for 5-HT_1B_, α-adrenergic_2B_, D_2_, and D_5_) and a radiolabeled receptor-specific ligand. IC_50_ determinations were based on the percent binding inhibition of the radiolabeled ligand.

5-HT, 5-hydroxytryptamine; D, dopamine; DHE, dihydroergotamine mesylate; IC_50_, half-maximal inhibitory concentration.

## Discussion

DHE has a long history as an efficacious acute therapy for migraine, with the explanation for its efficacy being its broad receptor pharmacology (8, 12). An update in our understanding of how DHE may acutely treat migraine is appropriate, particularly as there have been advancements in receptor binding methodology and new DHE products are being added to the research and development pipeline. Here we report updated data on DHE receptor pharmacology using the gpcrMAX assay panel, a high-throughput screening assay of GPCR ligands employing ß-arrestin recruitment. DHE (10 μM) was screened for functional activity at 168 GPCRs, demonstrating agonist activity across multiple receptor classes, including α-adrenergic_2B_, CXCR7, D_2L/2S/5_, and 5-HT_1A/1B/2A/2C/5A_ receptors (Table 7). This contrasted with sumatriptan succinate (10 μM), which demonstrated agonist activity at 4 receptors within a single class, 5-HT_1B/1E/1F/5A_. DHE also demonstrated antagonist activity at 9 receptors, including α-adrenergic_1B/2A/2C_, AMY_2_, D_1/3/4/5_, and 5-HT_1F_ receptors, across several receptor classes. The concentration used for this assay was high compared to DHE plasma concentrations; however, it was an assay requirement to ensure no potential receptor interaction was missed. Based on results from the screening assay, further assessment of DHE binding at therapeutically relevant concentrations was performed at select GPCRs to add clinical context.

**Table 7:** Summary of DHE activity at assayed receptors and clinical significance.

Receptor family	Receptor(s)	DHE activity (screening)	DHE binding (radioligand, IC_50_ [nM])	Clinical significance
Adreno-ceptors	α-adrenergic_2B_	Agonist	2.8	**Tolerability** Antagonistic adrenergic activity may be related to dizziness that can accompany IV DHE use (12)
	α-adrenergic_1B/2A/2C_	Antagonist	N/A	
Calcitonin	AMY_2_	Antagonist	N/A	**Therapeutic benefits** Amylin is structurally and functionally similar to CGRP, and may play a role in migraine pathophysiology (35–37)
Chemokine	CXCR7	Agonist	N/A	**Additional notes** Radioligand binding assays reported here did not show DHE binding at concentrations <1.0 μM; therefore, agonist activity of DHE on CXCR7 is unlikely in clinically relevant conditions
Dopamine	D_2L/2S/5_	Agonist	D_2_: 0.47	**Tolerability** The D_2_ receptor has been associated with nausea and vomiting (2, 12, 38, 39), a common side effect of IV DHE (10, 40–43), which may in part be related to agonism of DHE at D_2_ receptors **Therapeutic benefits** Modulation of dopamine may impact migraine symptoms (44) **Additional notes** DHE activity at receptor D_5_ showed both agonist (57% activity) and antagonist (54% activity) profiles
	D_1/3/4/5_	Antagonist	D_5_: 370	
5-Hydroxy-tryptamine	5-HT_1A/1B/2A/2C/5A_	Agonist	5-HT_1B_: 0.58	**Tolerability** Absence of DHE binding at 5-HT_3_ is noteworthy, as its activation can also produce nausea and vomiting (45–48) **Therapeutic benefits** Role of serotonin, particularly the 5-HT_1B_ receptor, has long been implicated in migraine pathophysiology (2, 49, 50) Therapeutic action of DHE may be related in part to agonist activity at the 5-HT_1B_ (9, 51) and 5-HT_1A_ receptors (52) Prolonged binding to 5-HT_1B/1D_ receptors may be a possible mechanism of the sustained efficacy of DHE (51) **Additional notes** Agonism at the 5-HT_2A_ receptor may be relevant, given its vasoconstrictive properties and implications in medication overuse headache pathophysiology (53) Because 5-HT_1F_ receptor agonists show efficacy in acutely treating migraine (54, 55), antagonism of this receptor by DHE suggests the 5-HT_1F_ receptor may not contribute to its therapeutic mode of action
	5-HT_1F_	Antagonist	N/A	
	5-HT_3_	Not screened	>300	
	5-HT_4E_	Not screened	230	
	5-HT_1D_	Not screened	N/A	

5-HT, 5-hydroxytryptamine; AMY_2_, amylin 2; CGRP, calcitonin gene-related peptide; CXCR7, CXC chemokine receptor 7; D, dopamine; DHE, dihydroergotamine mesylate; IC_50_, half-maximal inhibitory concentration; IV, intravenous; N/A, not applicable.

The binding (IC_50_) and agonist activity at the 5-HT_1B_ receptor were expected, and align with previously published data (11, 12, 56–59). Evidence supporting a role for serotonin in migraine pathophysiology is extensive, and the 5-HT_1B_ receptor has long been implicated, notably in the setting of triptans, by exerting its therapeutic effect on migraine symptoms via mediation of vasoconstriction in cranial and cerebral arteries and inhibition of relevant neural pathways (2, 49, 50). Sustained efficacy of DHE up to 48 h has been reported (9), and a study by Kori and colleagues (51) determined that prolonged binding to 5-HT_1B/1D_ receptors may be a possible mechanism for the sustained efficacy of DHE when used to treat migraine acutely. The dissociation half-lives of DHE on human 5-HT_1B_ (DHE: 1.38 h; sumatriptan: 0.17 h) and 5-HT_1D_ (DHE: 1.28 h; sumatriptan: 0.09 h) were approximately 10 times longer than those of sumatriptan, and DHE bound to these receptors 8–14 h longer than did sumatriptan. Importantly, our data confirm strong binding of DHE at the 5-HT_1B_ receptor with clinically relevant doses, suggesting that the therapeutic action of DHE may be due, at least in part, to agonist activity at the 5-HT_1B_ receptor. In agreement with previous work (12), our data show that DHE displayed agonist activity at the 5-HT_1A_ receptor. Newman-Tancredi and colleagues (60) assessed binding and agonist efficacy of DHE using recombinant human 5-HT_1A_ receptors expressed in CHO cells and determined that DHE bound strongly and displayed high-efficacy agonism (ie, E_max_ ≥ 90% relative to 5-HT) at 5-HT_1A_ receptors at nanomolar concentrations. Work by Hanoun and colleagues (52) characterized the action of DHE at the 5-HT_1A_ receptor in the rat brain, determining that DHE and its metabolite have an inhibitory influence on neuronal excitability and may potentially reduce anxiety via partial agonism at the 5-HT_1A_ receptor.

The D_2_ receptor has been implicated in the pathophysiology of nausea and vomiting, which are frequent symptoms that accompany migraine (2, 12, 38, 39). Nausea is also a common side effect of IV DHE use and most likely associated with the high C_max_ observed with its use (12, 40–43); therefore, it was not surprising that this study demonstrated DHE agonism and strong binding at the D_2_ receptor. Pretreatment with an antiemetic is a well-established option for preventing the nausea and vomiting associated with IV DHE use (41, 43). Some DHE products have reported a low rate of nausea, which may be due to their lower peak concentrations, (25, 61) suggesting D_2_ agonism by DHE may not necessarily result in increased nausea. Absence of DHE binding at the 5-HT_3_ receptor up to 300 nM is another noteworthy finding of this study, as the activation of 5-HT_3_ receptors can also produce nausea and vomiting (45–48). Interestingly, Cook and colleagues demonstrated antagonist activity at the D_2_ receptor with concentrations equivalent to the C_max_ of IV DHE and inhaled DHE (12). These discrepancies with the current study may be the result of differences in methodology (ß-arrestin recruitment vs. GPCR Ca^2+^ influx screening), antagonist or agonist cutoff requirements, or concentrations of DHE used (12). Our findings of agonism at the D_2_ receptor align with what has been reported in the literature (57). Further, according to a recent cross-sectional study, modulating the dopaminergic system should be considered for migraine treatment, as 32.6% of individuals with migraine experienced dopaminergic symptoms (eg, yawning, somnolence, nausea) during an attack. Attacks in these individuals were of longer duration and were more disabling than attacks in individuals without dopaminergic symptoms (44). Lastly, our assays detected both an agonistic (57% receptor activity) and antagonistic (54% receptor activity) profile for D_5_. Whether this finding suggests that DHE modulates the D_5_ receptor in a complex manner or is a result of the high concentration of DHE utilized (10 μM) and/or the defined cutoffs for determining significant agonist/antagonist activity (>30% or 50% receptor activity relative to the known receptor agonist or antagonist, respectively), would need to be assessed further in the future.

Interestingly, our study showed that DHE has demonstrated antagonist activity at the AMY_2_ receptor, 1 of 3 receptors for amylin, a peptide that is structurally and functionally similar to CGRP (35, 36). Recently, a randomized clinical trial showed that a synthetic amylin analogue, pramlintide, can induce migraine-like attacks in patients with migraine (62). Moreover, a recent prospective study reported higher interictal plasma amylin levels in patients with chronic migraine compared to healthy controls (37). The canonical focus on CGRP in migraine expanded to include amylin when it was discovered that 2 s-generation gepants antagonized both the CGRP receptor and amylin 1 (AMY_1_) receptor, the latter of which has been shown to be stimulated by CGRP and amylin with equal potency *in vitro* (63–65). These studies highlight an underappreciated role for and clinical relevance of amylin in migraine pathophysiology. In our study, DHE antagonized the AMY_2_ receptor, a high-affinity receptor for amylin (66), which may be therapeutically and clinically relevant to patients with migraine with high levels of interictal amylin signaling. Whether DHE has antagonism at the AMY1 receptor is a limitation of the current study, as it was not investigated due to lack of availability in the gpcrMAX assay. In future studies, it would be interesting to further delineate the role and interaction of DHE, amylin receptors, and migraine pathophysiology.

Data presented here revealed agonist activity of 10 μM DHE at the α-adrenergic_2B_ receptor and strong binding of therapeutically relevant doses of DHE, which was an unexpected finding. According to the literature, DHE binds to α- and β-adrenergic receptors (11, 56, 57); however, Cook and colleagues contrastingly reported functional antagonism at the α-adrenergic_2B_ receptor in addition to α-adrenergic_1A_ and α-adrenergic_2A_ receptors at a DHE concentration correlating to C_max_ for IV DHE and low or absent adrenergic antagonism for the MAP0004 doses, citing antagonism at the α-adrenergic receptors as a possible mechanism for the dizziness that accompanies DHE use (12). Our data demonstrated DHE antagonism at α-adrenergic_1B,2A,2C_ receptors at the 10 μM concentration, aligning with the Cook study. Interestingly, there are reports of an association between vasopressor effects and activation of vascular α-adrenergic_1_ and α-adrenergic_2_ receptors. Early work by Roquebert and Grenié (67, 68) reported that DHE elicited vasopressor effects in pithed rats, which were mediated by partial agonist activity at the α-adrenergic_2_ receptor but not the α-adrenergic_1_ receptor; however, this study did not consider the strong binding DHE exhibits at the 5-HT_2A_ receptor, a finding not known at the time. Rivera-Mancilla and colleagues (68) assessed the vasopressor responses to DHE following α-adrenergic_1_ and α-adrenergic_2_ receptor antagonist administration in pithed rats pretreated with ritanserin, an antagonist with very strong binding at 5-HT_2A_ receptors and very weak binding at α-adrenergic_1_ and α-adrenergic_2_ receptors, to eliminate the possibility of vasoconstriction mediated by the 5-HT_2A_ receptor. Results showed that vasopressor responses were present following administration of DHE, which were inhibited by both α-adrenergic_1_ and α-adrenergic_2_ receptor antagonists, theorizing the involvement of α-adrenergic_1A,1B,1D_ and α-adrenergic_2A,2B,2C_ receptors. However, the binding of DHE to the α-adrenergic_2B_ receptor was lower than to the α-adrenergic_2A_ and α-adrenergic_2C_ receptors (68). González-Hernández and colleagues (69) also utilized a pithed rat model to demonstrate that DHE blocks vasodepressor sensory CGRPergic outflow via the activation of the α-adrenergic_2_ receptor and 5-HT_1B/1D_ receptors. These findings were further corroborated by Villalón and colleagues (70), who reported vasoconstrictive properties of DHE mediated primarily—although, importantly, not exclusively—by 5-HT_1B_ and α-adrenergic_2A/2C_ receptors in a canine model. Kalkman and colleagues (71) compared the vasoconstrictive effects of DHE and ergotamine in rat aorta, demonstrating that ergotamine contracted rat aorta and behaved as a partial 5-HT_2A_ receptor agonist, whereas DHE was an insurmountable 5-HT_2A_ receptor antagonist. Cook and colleagues also reported that DHE was an antagonist at the 5-HT_2A_ receptor with 5 μM of DHE and at a DHE concentration correlating to C_max_ for IV DHE (~0.091 μM), with limited antagonism or an absence of functional activity with 4 MAP0004 inhalations (~0.007 μM) and 2 MAP0004 inhalations (~0.002 μM), respectively, suggesting it was unlikely that the 5-HT_2A_ receptor mediated coronary contraction (12). In contrast, we report agonist activity of 10 μM DHE at the 5-HT_2A_ receptor, which was a surprising finding. DHE product labels warn of potential cardiovascular (CV) and peripheral ischemic events, possibly attributed to agonist activity at the 5-HT_1B_ receptor, which can cause vasoconstriction of coronary arteries (72–74). However, it has been shown that the vasoconstrictive effects induced by DHE are more pronounced in the meningeal arteries than in the coronary arteries, suggesting patients without CV disease may not have this limitation or contraindication (75), and some DHE products have not reported increases in blood pressure in clinical studies (76). In addition to its vasoconstrictive properties, the 5-HT_2A_ receptor has been implicated in medication overuse headache (MOH) pathophysiology (53), although DHE is not known to be associated with high rates of MOH in the clinic (77). Agonism at the 5-HT_2B_ receptor has been implicated in drug-induced valvular heart disease (78). While Cook and colleagues showed no 5-HT_2B_ agonism for both MAP0004 doses, but agonism for IV DHE (12), this receptor was not screened in our study.

Another surprising finding was antagonist activity of DHE at the 5-HT_1F_ receptor. The initial screening assay demonstrated strong antagonist activity (92%) using 10 μM of DHE; however, this antagonist activity was found to be somewhat limited when further assessment using therapeutically relevant doses of DHE demonstrated an IC_50_ of 149 nM, indicating weak binding. Ergotamine has also shown weak binding at the 5-HT_1F_ receptor (79). The 5-HT_1F_ receptor agonist, lasmiditan, has shown efficacy in acutely treating migraine in several clinical studies (54, 55). The literature has also shown that DHE binds to the 5-HT_1F_ receptor (11, 26, 56–58). Although the Cook study did not evaluate binding at the 5-HT_1F_ receptor to compare findings to our study (12), the limited antagonist activity of DHE at the 5-HT_1F_ receptor in the present study could suggest that DHE does not demonstrate efficacy through activity at this receptor or that the DHE-binding kinetics are biased to the β-arrestin signaling pathways (80, 81). High agonist activity at the CXCR7 receptor (83%) using 10 μM of DHE was another unexpected result during the initial screening assay. Meyrath and colleagues (82) recently reported findings that CXCR7 (currently known as ACKR3) is an atypical scavenge receptor for a wide variety of opioid peptides, reducing their availability for the classical opioid receptors. The radioligand binding assay in this study revealed that DHE did not exert activity at CXCR7 at concentrations <1.0 μM, suggesting it is unlikely that DHE is active at CXCR7 under clinically relevant conditions.

This study has some limitations. First, some receptors that are important in migraine pathophysiology, such as 5-HT_1D_ and AMY_1_, were not screened because a human cell line expressing the receptors was unavailable for the assay for technical reasons or lack of availability. Second, agonist and antagonist activity of DHE and sumatriptan succinate at GCPRs was evaluated only via ß-arrestin recruitment. Because GCPRs can signal through several pathways, utilizing a single signaling pathway in this current study, ß-arrestin, may result in incomplete detection of functional activity of DHE and/or sumatriptan succinate (83, 84). Further, there is the possibility of a biased signaling that can further confound results (83).

## Conclusion

Using a new methodology to screen against 168 GPCRs via high-throughput assay, the receptor binding work presented here provides an update to our understanding of DHE receptor pharmacology. Similar to what has been reported in the literature, DHE in this study demonstrated broad receptor pharmacology, binding at several receptors across receptor classes, including agonist activity at α-adrenergic_2B_, CXCR7, D_2/5_, and 5-HT_1A/1B/2A/2C/5A_ receptors, and antagonist activity at α-adrenergic_1B/2A/2C_, AMY_2_, D_1/3/4/5_, and 5-HT_1F_ receptors. The antimigraine efficacy of DHE may be explained by agonism and strong binding of therapeutic doses at the 5-HT_1B_ receptor (5-HT_1D_ was not available in the GPCR assay), as well as slow dissociation, whereas the side effect profile of DHE may be attributed to agonist activity at the D_2_, α-adrenergic_2B_, and 5-HT_2A_ receptors. The exact interplay between activation and inhibition of multiple receptor pathways in the migraine cycle, extending beyond individual attacks and embracing organ systems beyond the central nervous system, has yet to be fully elucidated.

## Data availability statement

The datasets presented in this article are not readily available because raw data may include IP information that is not readily available for distribution without NDAs in place. Requests to access the datasets should be directed to saurora@impelpharma.com.

## Ethics statement

Ethical approval was not required for the studies on humans in accordance with the local legislation and institutional requirements because only commercially available established cell lines were used.

## Author contributions

LM: Writing – original draft, Writing – review & editing, Conceptualization, Data curation, Formal analysis. PG: Writing – original draft, Writing – review & editing, Conceptualization. RV: Writing – original draft, Writing – review & editing, Conceptualization. SR: Writing – original draft, Writing – review & editing, Conceptualization. SS: Writing – original draft, Writing – review & editing, Conceptualization. SA: Conceptualization, Supervision, Writing – original draft, Writing – review & editing.
