# Supplementary material for: New characterization of dihydroergotamine receptor pharmacology in the context of migraine: utilization of a β-arrestin recruitment assay
Source: Front Neurol. 2023 Nov 21;14:1282846. doi: 10.3389/fneur.2023.1282846 (PMC10703426; doi:10.3389/fneur.2023.1282846)
Supplement: Supplementary file 1 [file Data_Sheet_1.docx]

New Characterization of Dihydroergotamine Receptor Pharmacology in the Context of Migraine: Utilization of a β-arrestin Recruitment Assay

**Lisa McConnachie, PhD^1^; Peter J. Goadsby, MD, PhD^2,3^; Robert E. Vann, PhD^4^; Sutapa Ray, PhD^4^; Stephen B. Shrewsbury, MB ChB;^5^ Sheena K. Aurora, MD^4^**

**Correspondence:** Sheena K Aurora: [saurora@impelpharma.com](mailto:saurora@impelpharma.com); sheaur@yahoo.com

**In vitro screening for functional receptor activity of DHE and sumatriptan succinate methods**

Samples were added to cells and incubated for 90 or 180 minutes at 37 °C or room temperature, depending on the specific receptor, as noted, with specific incubation protocols outlined in **Supplemental Table 1**. For antagonist activity, preincubation temperatures were performed at the temperatures specified in **Supplemental Table 1**, per target. Incubation with 6× EC_80_ agonist was determined by the assay manufacturer and selected in order to provide a challenge that was low enough to be below the saturation point, so as to not bias the results, while still at a high enough challenge concentration to yield a robust signal-to-noise ratio. Further information is available from Eurofins Discovery Services upon request.

**Data analysis of functional receptor activity**

DHE and sumatriptan succinate activity were analyzed using CBIS data analysis suite (ChemInnovation, CA).

Agonist Mode

For agonist mode assays, percentage activity was calculated using the following formula:

% Activity = 100% × (mean RLU of DHE − mean RLU of vehicle control) / (mean MAX control ligand − mean RLU of vehicle control)

Activation of GPCRs by a compound acting as an agonist will result in an increase in β-arrestin recruitment to the target GPCR. To determine if DHE was potentially acting as an agonist to activate the receptor and induce β-arrestin recruitment the following factors were considered:

1. Is the DHE activity >30%?
   1. If so, is the DHE mean RLU > baseline RLU + 3 × baseline SD?
   2. Conversely, is the baseline RLU < DHE mean RLU – 3 × SD?
2. Is the DHE mean RLU / baseline RLU > 2?

If #2 is true and DHE activity is significantly different in cases 1a) and 1b), then the interaction is potentially significant.

Antagonist Mode

For antagonist mode assays, percentage inhibition was calculated using the following formula:

% Inhibition = 100% × (1 − (mean RLU of DHE − mean RLU of vehicle control) / (mean RLU of EC_80_ control − mean RLU of vehicle control))

Inhibition of GPCR activation by a compound acting as an antagonist of ligand binding will result in a decrease in the reference’s ability to induce β-arrestin recruitment to the target GPCR*.* To determine if DHE is potentially acting as an antagonist to inhibit receptor activation the following factors were considered:

1. Is the % inhibition > 50%?
   1. If so, is the compound mean RLU < EC_80_ RLU – 3 × EC_80_ SD?
   2. Conversely, is the EC_80_ RLU > compound mean RLU + 3 × compound SD?

If the inhibition is >50% and the compound activity is significantly different in cases a) and b), then the interaction is potentially significant.

**Radioligand competition binding assays**

Specific temperatures and incubation times for each target receptor assay outlined in the primary methods were pre-established by the manufacturer, based on assay optimization.

**Supplemental Table 1. Incubation Temperature and Times for Each Receptor Included in the Screening (1)**

| **Family Name** | **Human Gene** | **Common Name** | **Assay Incubation Temperature** | **Assay Incubation Time** |
| --- | --- | --- | --- | --- |
| 5-Hydroxytryptamine receptors | *HTR1A* | 5-HT_1A_ receptor | 37 °C | 2 hours |
|  | *HTR1B* | 5-HT_1B_ receptor | 37 °C | 2 hours |
|  | *HTR1F* | 5-HT_1F_ receptor | 37 °C | 2 hours |
|  | *HTR2A* | 5-HT_2A_ receptor | 37 °C | 2 hours |
|  | *HTR2C* | 5-HT_2C_ receptor | 37 °C | 2 hours |
|  | *HTR5A* | 5-HT_5A_ receptor | 37 °C | 2 hours |
|  | *HTR1E* | 5-HT_1E_ receptor | 37 °C | 2 hours |
| Acetylcholine receptors | *CHRM1* | M_1_ receptor | 37 °C | 2 hours |
|  | *CHRM2* | M_2_ receptor | 37 °C | 2 hours |
|  | *CHRM3* | M_3_ receptor | RT | 2 hours |
|  | *CHRM4* | M_4_ receptor | 37 °C | 2 hours |
|  | *CHRM5* | M_5_ receptor | 37 °C | 2 hours |
| Adenosine receptors | *ADORA3* | A_3_ receptor | 37 °C | 2 hours |
| Adrenoceptors | *ADRA1B* | α-adrenergic_1B_ (α_1B_-adrenoceptor) | 37 °C | 2 hours |
|  | *ADRA2A* | α-adrenergic_2A_ (α_2A_-adrenoceptor) | RT | 2 hours |
|  | *ADRA2B* | α-adrenergic_2B_ (α_2B_-adrenoceptor) | 37 °C | 2 hours |
|  | *ADRA2C* | α-adrenergic_2C_ (α_2C_-adrenoceptor) | RT | 2 hours |
|  | *ADRB1* | β-adrenergic_1_ (β_1_-adrenoceptor) | 37 °C | 2 hours |
|  | *ADRB2* | β-adrenergic_2_ (β_2_-adrenoceptor) | 37 °C | 2 hours |
| Angiotensin receptor | *AGTR1* | AT_1_ receptor | RT | 2 hours |
| Apelin receptor | *AGTRL1(APLNR)* | APJ (Apelin receptor) | RT | 2 hours |
| Bombesin receptors | *BRS3* | BB_3_ receptor | 37 °C | 2 hours |
|  | *GRPR* | BB_2_ receptor | 37 °C | 2 hours |
|  | *NMBR* | BB_1_ receptor | RT | 2 hours |
| Bradykinin receptors | *BDKRB1* | B_1_ receptor | RT | 2 hours |
|  | *BDKRB2* | B_2_ receptor | 37 °C | 2 hours |
| Calcitonin receptors | *CALCR* | CT receptor | 37 °C | 2 hours |
|  | *CALCRL-RAMP1 (NA)* | CGRP receptor | RT | 2 hours |
|  | *CALCRL-RAMP2 (NA)* | AM_1_ receptor | 37 °C | 2 hours |
|  | *CALCRL-RAMP3 (NA)* | AM_2_ receptor | RT | 2 hours |
|  | *CALCR-RAMP2*  *(NA)* | AMY_2_ receptor | 37 °C | 2 hours |
|  | *CALCR-RAMP3*  *(NA)* | AMY_3_ receptor | RT | 2 hours |
| Cannabinoid receptors | *CNR1* | CB_1_ receptor | 37 °C | 2 hours |
|  | *CNR2* | CB_2_ receptor | RT | 2 hours |
| Chemerin receptor | *CMKLR1* | CMKLR1 (Chemerin receptor 1) | 37 °C | 2 hours |
| Chemokine receptors | *CCR1* | CCR1 | 37 °C | 2 hours |
|  | *CCR10* | CCR10 | 37 °C | 2 hours |
|  | *CCR2* | CCR2 | 37 °C | 2 hours |
|  | *CCR3* | CCR3 | 37 °C | 2 hours |
|  | *CCR4* | CCR4 | 37 °C | 2 hours |
|  | *CCR5* | CCR5 | 37 °C | 2 hours |
|  | *CCR6* | CCR6 | 37 °C | 2 hours |
|  | *CCR7* | CCR7 | 37 °C | 2 hours |
|  | *CCR8* | CCR8 | 37 °C | 2 hours |
|  | *CCR9* | CCR9 | 37 °C | 2 hours |
|  | *CX_3_CR1* | CX_3_CR1 | 37 °C | 2 hours |
|  | *CXCR1* | CXCR1 | 37 °C | 2 hours |
|  | *CXCR2* | CXCR2 | 37 °C | 2 hours |
|  | *CXCR3* | CXCR3 | 37 °C | 2 hours |
|  | *CXCR4* | CXCR4 | 37 °C | 2 hours |
|  | *CXCR5* | CXCR5 | 37 °C | 2 hours |
|  | *CXCR7* | CXCR7 | 37 °C | 2 hours |
| Cholecystokinin receptors | *CCKAR* | CCK_1_ receptor | RT | 2 hours |
|  | *CCKBR* | CCK_2_ receptor | RT | 2 hours |
| Class A orphans | *EBI2 (GPR183)* | GPR183 | RT | 2 hours |
|  | *GPR1 (CMKLR2)* | GPR1 (Chemerin receptor 2) | 37 °C | 2 hours |
|  | *GPR119* | GPR119 | 37 °C | 2 hours |
|  | *GPR35* | GPR35 | 37 °C | 2 hours |
|  | *MRGPRX1* | MRGPRX1 | 37 °C | 2 hours |
|  | *MRGPRX2* | MRGX2 | 37 °C | 2 hours |
| Complement peptide receptors | *C5AR1* | C5A receptor (C5_a1_ receptor) | RT | 2 hours |
|  | *C5L2 (C5AR2)* | C5L2 receptor (C5_a2_ receptor) | 37 °C | 2 hours |
| Corticotropin-releasing factor receptors | *CRHR1* | CRF1 receptor | 37 °C | 2 hours |
|  | *CRHR2* | CRF2 receptor | RT | 2 hours |
| Dopamine receptors | *DRD1* | D_1_ receptor | 37 °C | 2 hours |
|  | *DRD2L* | D_2L_ receptor | 37 °C | 2 hours |
|  | *DRD2S* | D_2S_ receptor | RT | 2 hours |
|  | *DRD3* | D_3_ receptor | 37 °C | 2 hours |
|  | *DRD4* | D_4_ receptor | 37 °C | 2 hours |
|  | *DRD5* | D_5_ receptor | 37 °C | 2 hours |
| Endothelin receptors | *EDNRA* | ET_A_ receptor | RT | 2 hours |
|  | *EDNRB* | ET_B_ receptor | 37 °C | 2 hours |
| Formylpeptide receptors | *FPR1* | FPR1 | 37 °C | 2 hours |
|  | *FPRL1 (FPR2)* | FPR2/ALX | 37 °C | 2 hours |
| Free fatty acid receptors | *FFAR1* | FFA1 receptor | 37 °C | 4 hours |
|  | *GPR120 (FFAR4)* | FFA4 receptor | 37 °C | 2 hours |
| Galanin receptors | *GALR1* | GALR_1_ receptor (GAL_1_ receptor) | 37 °C | 2 hours |
|  | *GALR2* | GALR_2_ receptor (GAL_2_ receptor) | 37 °C | 2 hours |
| Ghrelin receptor | *GHSR* | ghrelin receptor | 37 °C | 2 hours |
| Glucagon receptors | *GCGR* | glucagon receptor | RT | 2 hours |
|  | *GIPR* | GIP receptor | 37 °C | 2 hours |
|  | *GLP1R* | GLP-1 receptor | 37 °C | 2 hours |
|  | *GLP2R* | GLP-2 receptor | 37 °C | 2 hours |
|  | *SCTR* | secretin receptor | 37 °C | 2 hours |
| Glycoprotein hormone receptors | *FSHR* | FSHR receptor (FSH receptor) | 37 °C | 2 hours |
|  | *LHCGR* | LH receptor | 37 °C | 2 hours |
|  | *TSHR(L) (TSHR)* | TSH receptor | RT | 4 hours |
| Histamine receptors | *HRH1* | H_1_ receptor | RT | 2 hours |
|  | *HRH2* | H_2_ receptor | RT | 2 hours |
|  | *HRH3* | H_3_ receptor | 37 °C | 2 hours |
|  | *HRH4* | H_4_ receptor | 37 °C | 4 hours |
| Hydroxycarboxylic acid receptors | *GPR109A (HCAR2)* | HCA_2_ receptor | 37 °C | 2 hours |
|  | *GPR109B (HCAR3)* | HCA_3_ receptor | 37 °C | 2 hours |
| Kisspeptin receptor | *KISS1R* | kisspeptin receptor | 37 °C | 2 hours |
| Leukotriene receptors | *LTB4R* | BLT_1_ receptor | 37 °C | 2 hours |
|  | *OXER1* | OXE receptor | RT | 2 hours |
| Lysophospholipid (LPA) receptors | *EDG4 (LPAR2)* | LPA_2_ receptor | RT | 3 hours |
|  | *EDG7 (LPAR3)* | LPA_3_ receptor | RT | 3 hours |
|  | *GPR92 (LPAR5)* | GPR92 receptor (LPA_5_ receptor*)* | RT | 3 hours |
| Lysophospholipid (S1P) receptors | *EDG1 (S1PR1)* | S1P_1_ receptor | 37 °C | 2 hours |
|  | *EDG3 (S1PR3)* | S1P_3_ receptor | 37 °C | 2 hours |
|  | *EDG5 (S1PR2)* | S1P_2_ receptor | 37 °C | 2 hours |
|  | *EDG6 (S1PR4)* | S1P_4_ receptor | 37 °C | 4 hours |
| Melanin-concentrating hormone receptors | *MCHR1* | MCH_1_ receptor | 37 °C | 2 hours |
|  | *MCHR2* | MCH_2_ receptor | 37 °C | 2 hours |
| Melanocortin receptors | *MC1R* | MC_1_ receptor | 37 °C | 2 hours |
|  | *MC3R* | MC_3_ receptor | 37 °C | 2 hours |
|  | *MC4R* | MC_4_ receptor | 37 °C | 2 hours |
|  | *MC5R* | MC_5_ receptor | 37 °C | 2 hours |
| Melatonin receptor | *MTNR1A* | MT_1_ receptor | 37 °C | 2 hours |
| Motilin receptor | *MLNR* | motilin receptor | 37 °C | 2 hours |
| Neuromedin U receptor | *NMU1R* | NMU1 receptor | 37 °C | 2 hours |
| Neuropeptide B and W receptors | *NPBWR1* | NPBW1 receptor | RT | 2 hours |
|  | *NPBWR2* | NPBW2 receptor | 37 °C | 2 hours |
| Neuropeptide FF and AF receptor | *NPFFR1* | NPFF1 receptor | 37 °C | 2 hours |
| Neuropeptide S receptor | *NPSR1b (NPSR1)* | NPS receptor | 37 °C | 2 hours |
| Neuropeptide Y receptors | *NPY1R* | Y_1_ receptor | RT | 2 hours |
|  | *NPY2R* | Y_2_ receptor | 37 °C | 2 hours |
|  | *PPYR1 (NPY4R)* | Y_4_ receptor | 37 °C | 2 hours |
| Neurotensin receptors | *NTSR1* | NTS_1_ receptor | 37 °C | 2 hours |
| Opioid receptors | *OPRD1* | δ receptor | RT | 2 hours |
|  | *OPRK1* | κ receptor | 37 °C | 2 hours |
|  | *OPRL1* | NOP receptor | RT | 2 hours |
|  | *OPRM1* | μ receptor | RT | 2 hours |
| Orexin receptors | *HCRTR1* | OX_1_ receptor | 37 °C | 2 hours |
|  | *HCRTR2* | OX_2_ receptor | 37 °C | 2 hours |
| P2Y receptors | *P2RY1* | P2Y_1_ receptor | RT | 2 hours |
|  | *P2RY11* | P2Y_11_ receptor | 37 °C | 2 hours |
|  | *P2RY12* | P2Y_12_ receptor | RT | 2 hours |
|  | *P2RY2* | P2Y_2_ receptor | 37 °C | 2 hours |
|  | *P2RY4* | P2Y_4_ receptor | 37 °C | 2 hours |
|  | *P2RY6* | P2Y_6_ receptor | 37 °C | 2 hours |
| Parathyroid hormone receptors | *PTHR1 (PTH1R)* | PTH1 receptor | 37 °C | 2 hours |
|  | *PTHR2 (PTH2R)* | PTH2 receptor | 37 °C | 2 hours |
| Peptide P518 receptor | *GPR103 (QRFPR)* | QRFPR receptor | RT | 2 hours |
| Platelet-activating factor receptor | *PTAFR* | PAF receptor | 37 °C | 2 hours |
| Prokineticin receptors | *PROKR1* | PKR_1_ receptor | 37 °C | 2 hours |
|  | *PROKR2* | PKR_2_ receptor | 37 °C | 2 hours |
| Prolactin-releasing peptide receptor | *PRLHR* | PRRP receptor (PrRP receptor) | 37 °C | 2 hours |
| Prostanoid receptors | *CRTH2 (PTGDR2)* | PTGDR2 receptor (DP_2_ receptor) | RT | 2 hours |
|  | *PTGER2* | EP_2_ receptor | 37 °C | 2 hours |
|  | *PTGER3* | EP_3_ receptor | 37 °C | 2 hours |
|  | *PTGER4* | EP_4_ receptor | 37 °C | 2 hours |
|  | *PTGFR* | FP receptor | 37 °C | 2 hours |
|  | *PTGIR* | IP1 receptor (IP receptor) | 37 °C | 2 hours |
|  | *TBXA2R* | TP receptor | 37 °C | 2 hours |
| Protease activated receptors | *F2R* | PAR1 | RT | 2 hours |
|  | *F2RL1* | PAR2 | RT | 2 hours |
|  | *F2RL3* | PAR4 | 37 °C | 2 hours |
| Relaxin family peptide receptor | *RXFP3* | RXFP3 | 37 °C | 2 hours |
| Somatostatin receptors | *SSTR1* | SST_1_ receptor | 37 °C | 2 hours |
|  | *SSTR2* | SST_2_ receptor | 37 °C | 2 hours |
|  | *SSTR3* | SST_3_ receptor | 37 °C | 2 hours |
|  | *SSTR5* | SST_5_ receptor | 37 °C | 2 hours |
| Tachykinin receptors | *TACR1* | NK_1_ receptor | 37 °C | 2 hours |
|  | *TACR2* | NK_2_ receptor | 37 °C | 2 hours |
|  | *TACR3* | NK_3_ receptor | 37 °C | 2 hours |
| Thyrotropin-releasing hormone receptor | *TRHR* | TRH_1_ receptor | 37 °C | 2 hours |
| Urotensin receptor | *UTR2 (UTS2R)* | UT receptor | RT | 2 hours |
| Vasopressin and oxytocin receptors | *AVPR1A* | V_1A_ receptor | RT | 2 hours |
|  | *AVPR1B* | V_1B_ receptor | RT | 2 hours |
|  | *AVPR2* | V_2_ receptor | RT | 2 hours |
|  | *OXTR* | OT receptor | 37 °C | 2 hours |
| VIP and PACAP receptors | *ADCYAP1R1* | PAC_1_ receptor | 37 °C | 2 hours |
|  | *VIPR1* | VPAC_1_ receptor | RT | 2 hours |
|  | *VIPR2* | VPAC_2_ receptor | 37 °C | 2 hours |

Note: This table refers to receptor nomenclature at the time of assay performance. Information in parentheses refers to any updates in nomenclatures per IUPHAR guidelines (2).

DHE = dihydroergotamine mesylate; IUPHAR = International Union of Basic and Clinical Pharmacology; LPA = lysophosphatidic acid; NA = not applicable; S1P = sphingosine-1 phosphate; PACAP = pituitary adenylate cyclase-activating peptide; RT = room temperature; VIP = vasoactive intestinal peptide.

1. Eurofins. GPCR Assay Formats and Services Offered. <https://www.discoverx.com/services/drug-discovery-development-services/gpcr-screening-profiling-services/gpcrscan-gpcr-profiling/gpcrmax>. Accessed October 27, 2022.

2. IUPHAR GPCR nomenclature. <https://www.guidetopharmacology.org/GRAC/GPCRListForward?class=A>. Accessed October 18, 2023.
